# An Adaptive Coding Pass Scanning Algorithm for Optimal Rate Control in Biomedical Images

**DOI:** 10.1155/2012/935914

**Published:** 2011-10-15

**Authors:** Hsi-Chin Hsin, Tze-Yun Sung, Yaw-Shih Shieh

**Affiliations:** ^1^Department of Computer Science and Information Engineering, National United University, Miaoli City 36003, Taiwan; ^2^Department of Electronics Engineering, Chung Hua University, Hsinchu City 30012, Taiwan

## Abstract

High-efficiency, high-quality biomedical image compression is desirable especially for the telemedicine applications. This paper presents an adaptive coding pass scanning (ACPS) algorithm for optimal rate control. It can identify the significant portions of an image and discard insignificant ones as early as possible. As a result, waste of computational power and memory space can be avoided. We replace the benchmark algorithm known as postcompression rate distortion (PCRD) by ACPS. Experimental results show that ACPS is preferable to PCRD in terms of the rate distortion curve and computation time.

## 1. Introduction

With the rapid growth of modern telemedicine techniques, high-efficiency image-processing algorithms are essentially in great demand [1, 2]. Wavelet transform (WT) [3–5] provides many desirable properties that are beneficial to the biomedical image applications. In general, the significant wavelet coefficients of an image are clustered within each subband; this property is the main idea behind the embedded block coding with optimized truncation (EBCOT) algorithm, which is the core of JPEG2000 [6, 7].

EBCOT adopts the post compression rate distortion (PCRD) algorithm for optimal rate control, which demands a large amount of memory space and leads to waste of computational power [8]. Du et al. utilized a fixed scan order to reduce the computation time [9], which however lacks adaptation. Vikram et al. proposed a scheme to estimate the rate distortion slopes of code blocks for adaptive rate control [10]. Fang et al. proposed a precompression scheme to avoid unneeded computations [11]. An et al. proposed a fractional upward shift for lossless image compression [12]. Auli-Llinas et al. proposed nonuniform adaptation for bit plane coding [13]. Balster et al. proposed inter-based adaptation to estimate the distortion value [14]. Xue et al. distributed the target bit rates adaptively to reduce the computational complexity [15].

This paper proposes an adaptive coding pass scanning (ACPS) algorithm to rearrange the coding passes of an image such that the most significant one has priority over others. In comparison with the complicated approach used in [10–15], which is either time consuming or hardware demanding, ACPS makes good use of the MQ table that is available at both encoder and decoder. As a result, there is no need to store and transmit any information about the contribution of coding passes, and it has the advantage of saving a lot of computation time, memory space and transmission time, which is preferable for many applications [16–20].

The remainder of this paper proceeds as follows. In Section 2, EBCOT is reviewed briefly.  Section 3 describes the proposed ACPS algorithm. Experimental results are presented in Section 4. Conclusion is given in Section 5.

## 2. Review of EBCOT

**Figure 1:**
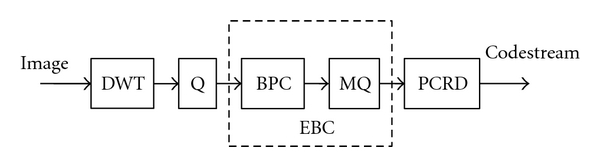
Block diagram of the EBCOT encoder.

The embedded block coding with optimized truncation (EBCOT) algorithm adopts wavelet transform for subband decomposition. It is a two-tier algorithm. Tier-1 performs bit plane coding (BPC) followed by arithmetic coding (AC). Tier-2 aims for optimal rate control.  Figure 1 depicts the block diagram of EBCOT encoder. Three coding passes, namely the significance propagation (SP) pass, the magnitude refinement (MR) pass and the cleanup (CU) pass are defined in EBCOT. For a wavelet coefficient that is currently insignificant, if any of the 8 neighboring coefficients are significant, it is coded in the SP pass using the significance coding operation; otherwise, it is coded in the CU pass using the cleanup coding operation. If the coefficient becomes significant, its sign is then coded using the sign coding operation. The significant coefficients are recursively updated using the magnitude refinement coding operation in the MR pass. The bit streams of the coding passes can be further compressed by AC with the probability models stored in the MQ table.

EBCOT applies the postcompression rate distortion (PCRD) algorithm for optimal rate control. Specifically, let {*B*_*i*_} be the code blocks of an image. *B*_*i*_ is coded from the most significant bit plane to the least significant bit plane, and terminates at some point, says *n*_*i*_, resulting in a bit rate denoted by *R*_*i*_^*n*_*i*_^. All of the end points of coding passes are possible truncation points. The distortion incurred by discarding the coding passes after *n*_*i*_ is denoted by *D*_*i*_^*n*_*i*_^. PCRD selects optimal truncation points by minimizing the total distortion given by

(1) $$D=\sum_{i}D_{i}^{n_{i}},$$

with the following rate constraint:

(2) $$R=\sum_{i}R_{i}^{n_{i}}\leq R_{c},$$

where *R*_*c*_ is the target bit rate. As all of the code blocks including insignificant ones must be coded before PCRD, the drawbacks are wastes of computational power, execution time, and memory space.

## 3. Image Coding with Adaptive Coding Pass Scanning

For the telemedicine applications, the following question is often raised. Is there useful information available at both encoder and decoder based on which the contribution of coding passes can be obtained? If so, there is no need to transmit the scanning order of coding passes, from encoder to decoder, for optimal rate control. Motivated by the MQ coder, we propose a novel scheme to estimate the rate distortion slope (RDS) of coding passes. Only the coding passes with significant RDS are used to generate the final code stream, and the unneeded ones with insignificant RDS can be discarded before EBCOT tier-1. Thus, it has the advantage of saving computational power, memory space, and transmission time. 

### 3.1. Adaptive Coding Pass Scanning Algorithm

In [16], we proposed the CBRDE algorithm to arrange the code blocks of an image adaptively. Specifically, let *b*_*ij*_(*m*, *n*) be a binary function of position (*m*, *n*) in the *i*th code block, at the *j*th bit plane, and *B*_*ij*_ = ⋃_*m*,*n*_{*b*_*ij*_(*m*, *n*)}. The RDS of *B*_*ij*_ is defined as

(3) $$\begin{array}{c} S_{ij}=\frac{E\left[\Delta D_{ij}\right]}{E\left[\Delta R_{ij}\right]},\\ \;\text{where}\;E\left[\Delta D_{ij}\right]=\sum_{m,n}\text{prob}\left(b_{ij}\left(m,n\right)=1\right),\\ \;E\left[\Delta R_{ij}\right]=\sum_{m,n}H\left(b_{ij}\left(m,n\right)\right),\\ H\left(b\right)=-\text{prob}\left(b=1\right)\cdot \text{lo}{\text{g}}_{2}\left(\text{prob}\left(b=1\right)\right)\\ \;\;\;\;-\text{prob}\left(b=0\right)\cdot \text{lo}{\text{g}}_{2}\left(\text{prob}\left(b=0\right)\right),\\ \text{prob}\left(b_{ij}\left(m,n\right)=1\right)=\{\begin{array}{cc} Qe\_\text{Value}, & \text{if}\;\text{MPS}=0,\\ 1-Qe\_\text{Value}, & \text{if}\;\text{MPS}=1, \end{array} \end{array}$$

Δ*D*_*ij*_ and Δ*R*_*ij*_ are the distortion decreased and the coding bits increased for *B*_*ij*_, respectively, *E*(∘) is the expectation operation, *H*(∘) is the entropy operation, and *Qe*_Value is the probability of less probable symbol (LPS), which can be obtained from the MQ table directly.

**Figure 2:**
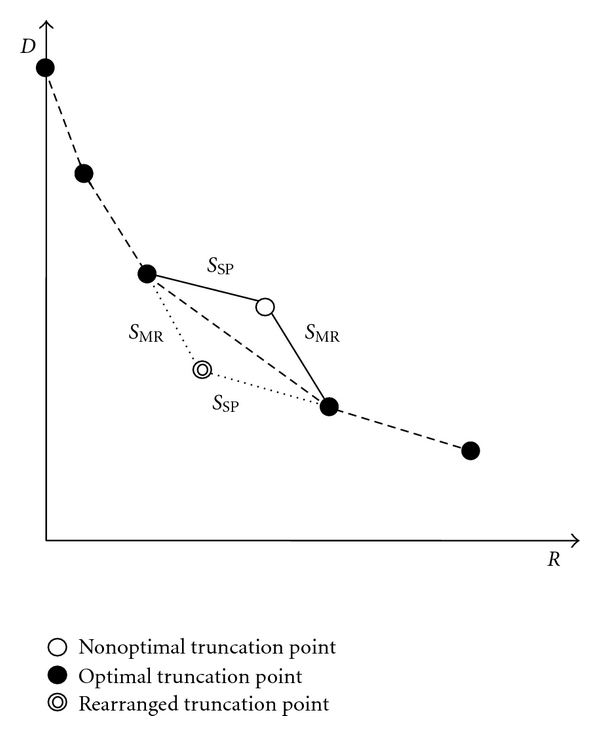
RD curves formed by nonoptimal truncation point (empty circle), optimal truncation points (dark circles), and rearranged truncation point (double-ring circle) *S*_SP_ and *S*_MR_ are the RDS of the SP and MR passes in a code block.

**Figure 3:**
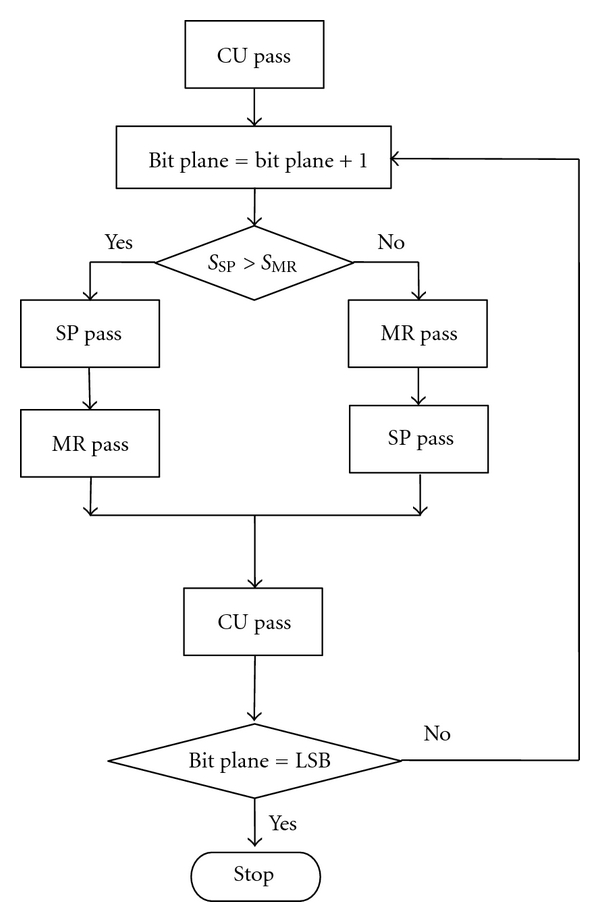
Flowchart of the proposed ACPS algorithm.

At each bit plane, each code block is coded with a sequence of three coding passes, that is, SP followed by MR and CU. All of the coding passes are possible truncation points. It is noted that more than 75% of the coding passes of an image are optimal truncation points, and more than 97% of which are either consecutive or one coding pass away from each other [21]. Motivated by the above, the adaptive coding pass scanning (ACPS) algorithm is thus proposed. As shown in Figure 2, if the RDS of the MR pass is greater than that of the SP pass, the MR pass should be performed immediately after the SP pass according to the PCRD algorithm, and the optimal segment should contain the SP and MR passes. However, unlike the CU pass, the MR pass may take place before the SP pass at each bit plane. In order to improve the compression performance, the above example is modified as follows. The scan order of SP and MR passes is rearranged such that the one with greater RDS should be coded first, which leads to the ACPS algorithm.  Figure 3 depicts the flowchart of ACPS, in which CBRDE is used to estimate the RDS of coding passes.

### 3.2. Image Coder with ACPS

**Figure 4:**
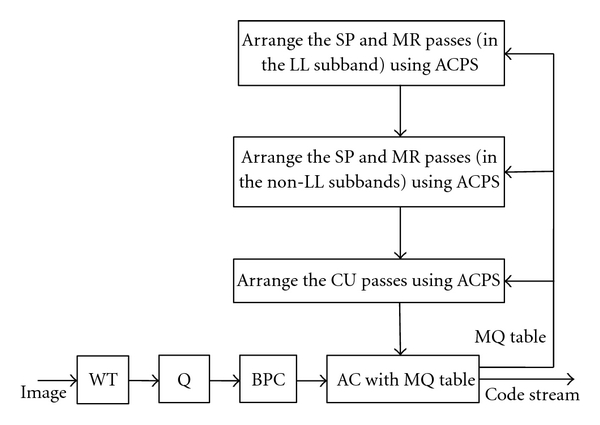
Block diagram of the proposed image encoder with ACPS.

**Figure 5:**
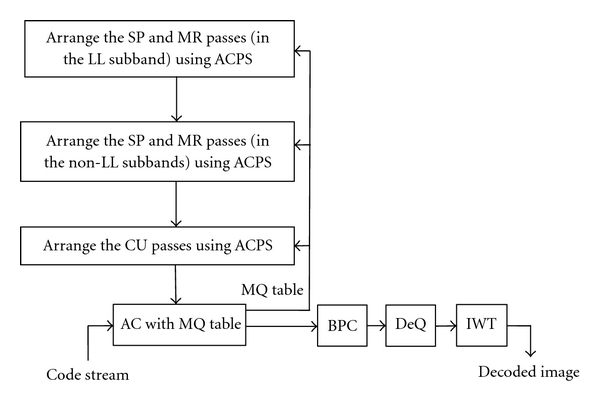
Block diagram of the proposed image decoder with ACPS.

As a large amount of energy is in the LL subband, it should be coded first. For the coding passes of the non-LL subbands, the one with the greatest RDS should be coded as early as possible. Hence, we propose an image coder with ACPS. Block diagrams of the proposed encoder and decoder are shown in Figures 4 and 5, respectively. The encoding steps are as follows.

**Step 1**

Initialization. Decompose an image into wavelet subbands, quantize the transform coefficients, and divide each subband into code blocks. Initially, the fixed scan order [21] is used. More specifically, wavelet subbands are scanned in a zigzag order, from the lowest frequency subband to the highest frequency subband, the code blocks within each subband are scanned from left to right, top to bottom, the first coding pass for each code block is the CU pass, and the scan order at the next bit plane is the SP pass followed by the MR pass and the CU pass.

**Step 2**

Estimation of RDS. At the current bit plane, estimate the RDS of coding passes by CBRDE. ACPS is then used to rearrange the scan order of coding passes.

**Step 3**

Adaptive Scan of Coding Passes. As the LL subband contains lots of significant coefficients, the SP and MR passes in the LL subband are a top priority. All the SP and MR passes in the non-LL subbands have priority over the CU passes at the current bit plane.

**Step 4**

EBCOT tier-1 Encoding. After rearranging the coding passes at the current bit plane, EBCOT tier-1 is used to produce the code streams that can be transmitted to decoder immediately. For the next bit plane, go to step 2, and continue the steps in order. Terminate at any point while the accumulated bit rate reaches the target bit rate.

**Figure 6:**
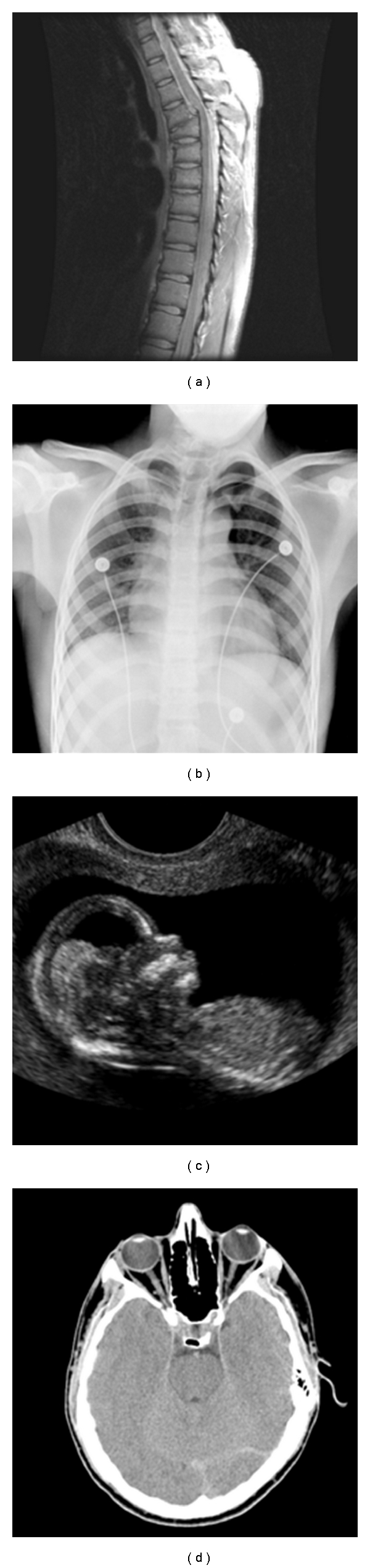
Test images; (a) Spine (MRI), (b) Chest (X-ray), (c) Fetus (ultrasonic), and (d) Head (CT).

To show the potential of ACPS, the following correct rate is utilized. Let the SP, MR, and CU passes in the *i*th code block at the *j*th bit plane be represented as *C*_*ij*_^0^, *C*_*ij*_^1^, and *C*_*ij*_^2^, respectively. $\hat{P}(C_{ij}^{k})$ and *P*(*C*_*ij*_^*k*^) denote the location indices of *C*_*ij*_^*k*^, *k* ∈ {0,1, 2} for the estimated scan order and the true scan order, respectively. We measure the correct rate of ACPS below:

(4) $$CR_{j}=(1-\frac{1}{N_{j}^{2}}\sum_{i}\overset{2}{\underset{k=0}{\sum }}\left|\hat{P}\left(C_{ij}^{k}\right)-P\left(C_{ij}^{k}\right)\right|)\times 100\text{\%},$$

where *N*_*j*_ is the total number of coding passes at the *j*th bit plane. The results of images shown in Figure 6 are given in Table 1. It is noted that the correct rates for the first two bit planes are extremely high due to the suitable initialization using [21]. For the first few bit planes after the non-LL subbands become significant, the correct rates are less than 70%, because the MQ table is not quite reliable due to only a small number of significant coefficients appearing. However, the correct rates become higher as the MQ table becomes more reliable. The overall correct rate is approximately 80%. It justifies the soundness of ACPS.

## 4. Experimental Results in Biomedical Image Processing

The proposed image coder with ACPS has been evaluated on various biomedical images. The experimental results of biomedical images shown in Figure 6, namely, spine, chest, fetus, and head, are given in this paper, which are representative of magnetic resonance image (MRI), X-ray, ultrasound, and computed tomography (CT) images, respectively. The 9/7-wavelet filters adopted by the JPEG2000 standard are used. The distortion is defined as the peak-signal-to-noise ratio (PSNR), together with the rate of bits per pixel (bpp) forms the rate distortion (RD) curve.

**Figure 7:**
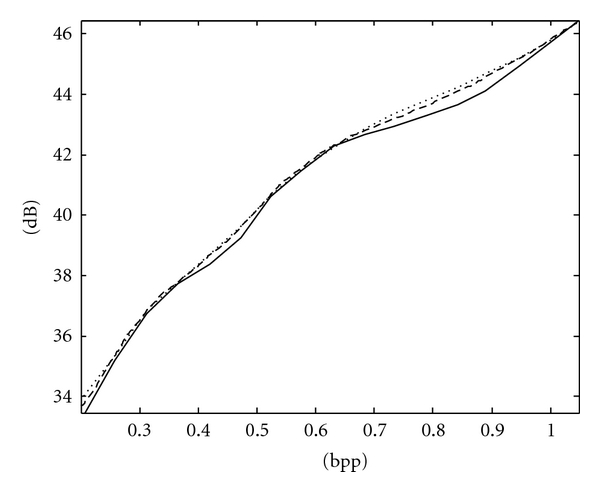
RD curves of Spine MRI image using the fixed scan order [21] (solid line), PCRD (dashed line), and ACPS (dotted line).

**Figure 8:**
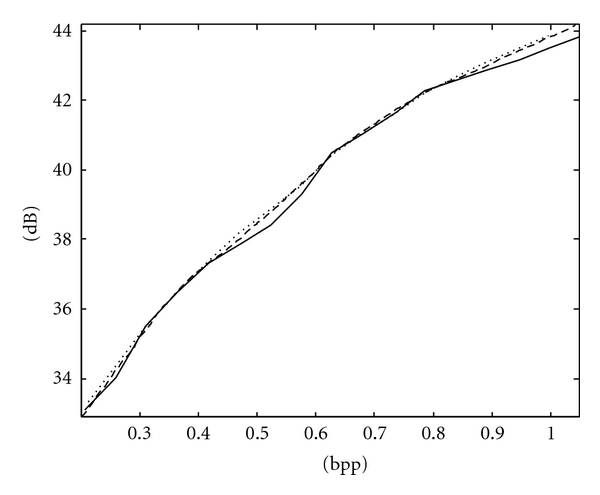
RD curves of Chest X-ray image using the fixed scan order [21] (solid line), PCRD (dashed line), and ACPS (dotted line).

**Figure 9:**
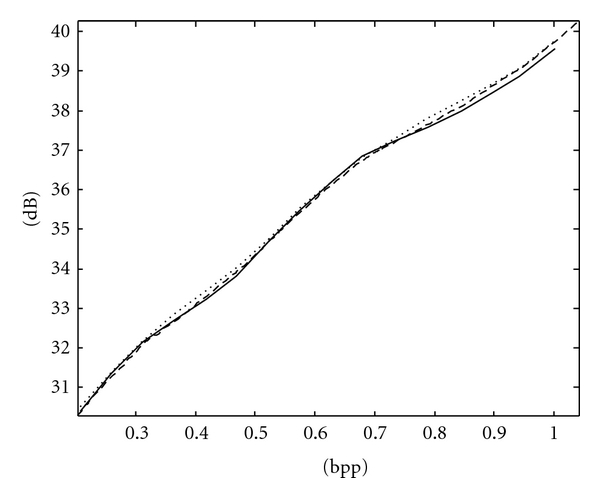
RD curves of Fetus ultrasonic image using the fixed scan order [21] (solid line), PCRD (dashed line), and ACPS (dotted line).

**Figure 10:**
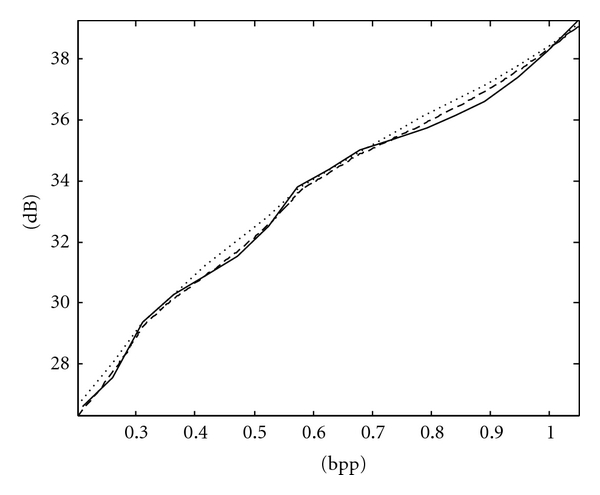
RD curves of Head CT image using the fixed scan order [21] (solid line), PCRD (dashed line), and ACPS (dotted line).

The RD curves of Spine and Chest images using ACPS and PCRD are comparable and more convex than that using the fixed scan order [21], as shown in Figures 7 and 8, respectively, where the horizontal and vertical axes are the bpp rates and PSNR values. For fetus and head images, ACPS is preferable to both PCRD and the fixed scan order, as shown in Figures 9 and 10, respectively.

Since ACPS takes the place of PCRD, lots of insignificant coding passes can be discarded as early as possible, waste of computation time and memory space can be avoided, and a great improvement in speed results. As shown in Table 2, the speed-up ranges from 30% to 50%.

## 5. Conclusion

In EBCOT, the code blocks of an image are independently coded with three coding passes, that is, SP followed by MR and CU passes. Any coding passes are possible truncation points, based on which, optimal truncation points can be obtained by using the PCRD algorithm. It requires that all the coding passes are coded and stored in the tier-1 of EBCOT; however, lots of insignificant coding passes are not needed, which in turn are discarded after PCRD. Instead of computing the true RDS, ACPS makes use of the estimated RDS. It is based on the MQ table, which is available at both encoder and decoder; thus, there is no need to transmit the scan order of the significant coding passes. Experimental results in biomedical image processing show that a great improvement in speed can be obtained by replacing PCRD with ACPS.

## Figures and Tables

**Table 1:** The correct rates of ACPS.

Image	Spine	Chest	Fetus	Head
Bit-plane 1	100%	100%	100%	100%
Bit-plane 2	100%	100%	100%	100%
Bit-plane 3	66.7%	66.7%	66.7%	63.7%
Bit-plane 4	70.3%	70.3%	74.5%	78.5%
Bit-plane 5	85.1%	71.9%	79.2%	69.5%
Bit-plane 6	70.4%	74.9%	78.5%	73.9%
Bit-plane 7	73.4%	78.3%	77.9%	76.7%
Bit-plane 8	79.2%	74.3%	73.1%	77.9%

**Table 2:** Improvement in speed using ACPS compared to PCRD.

Image	Speed up
Spine (MRI)	32.7%
Chest (X-ray)	33.1%
Fetus (ultrasonic)	52.7%
Head (CT)	53.6%
